# Whole Genome Sequencing Reveals Antimicrobial Resistance and Virulence Genes of Both Pathogenic and Non-Pathogenic *B. cereus* Group Isolates from Foodstuffs in Thailand

**DOI:** 10.3390/antibiotics13030245

**Published:** 2024-03-07

**Authors:** Phornphan Sornchuer, Kritsakorn Saninjuk, Sumet Amonyingcharoen, Jittiporn Ruangtong, Nattaya Thongsepee, Pongsakorn Martviset, Pathanin Chantree, Kant Sangpairoj

**Affiliations:** 1 Department of Preclinical Science, Faculty of Medicine, Thammasat University, Pathum Thani 12120, Thailand; nattayat@tu.ac.th (N.T.); pong_m@tu.ac.th (P.M.); pathanin@tu.ac.th (P.C.); kant_san@tu.ac.th (K.S.); 2 Thammasat University Research Unit in Nutraceuticals and Food Safety, Faculty of Medicine, Thammasat University, Pathum Thani 12120, Thailand; jittiporn.rt@gmail.com; 3 Porcinotec Co., Ltd., Nonthaburi 11000, Thailand; kritsakorn.s@porcinotec.com; 4 Medical Life Sciences Institute, Department of Medical Sciences, Ministry of Public Health, Nonthaburi 11000, Thailand; sumet.a@dmsc.mail.go.th

**Keywords:** *Bacillus cereus*, foodborne pathogen, antimicrobial resistance gene, virulence gene, whole-genome sequencing

## Abstract

Members of the *Bacillus cereus* group are spore-forming Gram-positive bacilli that are commonly associated with diarrheal or emetic food poisoning. They are widespread in nature and frequently present in both raw and processed food products. Here, we genetically characterized 24 *B. cereus* group isolates from foodstuffs. Whole-genome sequencing (WGS) revealed that most of the isolates were closely related to *B. cereus sensu stricto* (12 isolates), followed by *B. pacificus* (5 isolates), *B. paranthracis* (5 isolates), *B. tropicus* (1 isolate), and “*B. bingmayongensis*” (1 isolate). The most detected virulence genes were *BAS_RS06430*, followed by bacillibactin biosynthesis genes (*dhbA*, *dhbB*, *dhbC*, *dhbE*, and *dhbF*), genes encoding the three-component non-hemolytic enterotoxin (*nheA*, *nheB*, and *nheC*), a gene encoding an iron-regulated leucine-rich surface protein (*ilsA*), and a gene encoding a metalloprotease (*inhA*). Various biofilm-associated genes were found, with high prevalences of *tasA* and *sipW* genes (matrix protein-encoding genes); *purA*, *purC*, and *purL* genes (eDNA synthesis genes); *lytR* and *ugd* genes (matrix polysaccharide synthesis genes); and *abrB*, *codY*, *nprR*, *plcR*, *sinR*, and *spo0A* genes (biofilm transcription regulator genes). Genes related to fosfomycin and beta-lactam resistance were identified in most of the isolates. We therefore demonstrated that WGS analysis represents a useful tool for rapidly identifying and characterizing *B. cereus* group strains. Determining the genetic epidemiology, the presence of virulence and antimicrobial resistance genes, and the pathogenic potential of each strain is crucial for improving the risk assessment of foodborne *B. cereus* group strains.

## 1. Introduction

*Bacillus* species, which are spore-forming Gram-positive bacteria, are widespread in nature as spores and vegetative cells. They easily spread to both raw and processed food such as rice, meat, seafood, vegetables, and dairy products. As the spores are resistant to heat, freezing, drying, and radiation, there is a risk of their transmission in heat-treated and processed food products.

The *B. cereus* group, also known as *B. cereus sensu lato* (*s.l.*), consists of three main species: *B. cereus sensu stricto* (*s.s.*), which is an opportunistic pathogen associated with foodborne infections as well as extraintestinal infections; *B. anthracis*, the etiological agent of anthrax in humans and animals; and *B. thuringiensis*, an entomopathogen that is well-known for its use as a biopesticide [1,2]. The *B. cereus* group also includes *B. mycoides* and *B. pseudomycoides*, which are characterized by rhizoidal colonies on culture media [3]; *B. weihenstephanensis* and *B. wiedmannii*, which are psychrotolerant bacteria [4,5,6]; and *B. cytotoxicus*, which is a thermotolerant bacterium [7]. Other bacteria that are classified as *B. cereus* group species are *B. albus*, *B. bingmayongensis*, *B. fungorum*, *B. gaemokensis*, *B. luti*, *B. manliponensis*, *B. mobilis*, *B. nitratireducens*, *B. pacificus*, *B. paramycoides*, *B. paranthracis*, *B. proteolyticus*, *B. toyonensis*, and *B. tropicus* [8,9,10,11]. Additionally, there are more species recently confirmed to be members of this group according to the List of Prokaryotic Names with Standing in Nomenclature (LPSN; http://www.bacterio.net, accessed on 10 February 2024).

Some members of the *B. cereus* group are causative agents of gastrointestinal syndrome (emetic type or diarrheal type) and non-gastrointestinal tract infections, including septicemia, central nervous system infection, respiratory tract infection, and severe ocular infection, especially in immunocompromised individuals [12,13]. The pathogenicity of *B. cereus*, whether intestinal or non-intestinal, is associated with the production of tissue-destructive exotoxins (hemolysins, phospholipases, pore-forming enterotoxins) and emesis-inducing toxin [14,15]. The notable pore-forming enterotoxins comprise hemolysin BL (HBL), non-hemolytic enterotoxin (NHE), and cytotoxin K (CytK) [16,17,18]. The enterotoxins are produced by vegetative cells (which are ingested as viable cells or spores) in the small intestine, which causes abdominal pain and watery diarrhea [15]. The emesis-inducing toxin cereulide, a plasmid-encoded cyclic peptide, can be synthesized in contaminated food products such as rice, milk, and pasta. Other virulence factors include hemolysin II (encoded by *hlyII*), enterotoxin FM (encoded by *entFM*), and another putative enterotoxin (encoded by *entABC*) [19].

Biofilm formation can be associated with chronic and/or severe bacterial infections in humans as it can protect bacteria against antimicrobials and/or the host immune system. Biofilms are also important in the food industry, especially for the *B. cereus* group where biofilms can contaminate food products. Biofilm is considered an extremely strong extracellular matrix, and it facilitates bacterial attachment to both abiotic and biotic surfaces [20]. Moreover, biofilm formation mediates the development and transmission of antimicrobial resistance (AMR) genes by facilitating bacterial interactions within the biofilm [21,22]. Therefore, biofilm formation is considered to be an important virulence determinant of various pathogenic bacteria including *Bacillus* species [23].

Although most *B. cereus* group infections may not need antimicrobial treatment, it is still crucial to prepare for some circumstances, especially those that warrant critical care [24]. Foodborne illness associated with *B. cereus* group strains do not need to be treated with antibiotics. However, *B. cereus* bacteremia, which can cause severe life-threatening systemic infections especially in immunocompromised patients, need to be treated with appropriate antibiotic agents. Extensive antimicrobial use can lead to the emergence of AMR bacterial strains, which can cause the failure of routine treatments. Determining the AMR profile of *Bacillus* species is important not only for treatment selection but also for providing basic knowledge on the transmissible AMR genes in the food chain.

The number of whole-genome sequencing (WGS) studies of bacteria has increased as the cost of second-generation, short-read sequencing platforms (e.g., Illumina) has steadily decreased [25]. WGS is an effective strategy for rapidly characterizing *B. cereus* group strains, in terms of their genetic epidemiology and the presence of virulence and AMR genes. The use of genetic loci as markers to discriminate between pathogenic and nonpathogenic *B. cereus* group strains is more effective than phenotypic and biochemical methods in some circumstances [26,27]. The pantoate-beta-alanine ligase (*panC*) gene is used to classify *B. cereus* isolates (e.g., from the environment and cases of bacteremia in humans) into seven phylogenetic clades (I to VII) [10,28].

In this study, 24 *B. cereus* group isolates were characterized by WGS using Illumina sequencing. The presence of genes related to virulence and AMR was evaluated in all isolates. The study aimed to characterize these foodborne bacteria, which may cause health hazards if not controlled.

## 2. Results

### 2.1. Phylogenetic Analysis of B. cereus Group Isolates

**Figure 1:**
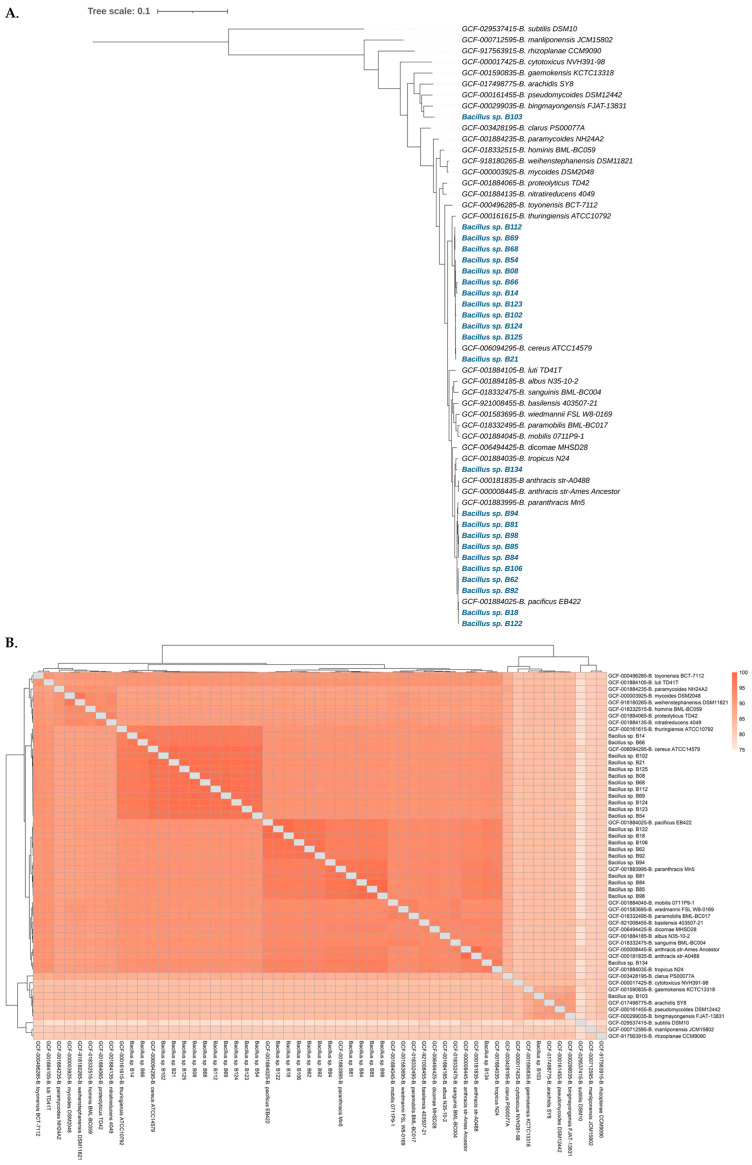
(**A**) Maximum likelihood phylogenetic tree of the 24 *B. cereus* group isolates (in blue font) and other type strains of *B. cereus* group genome sequences, generated using the GToTree v1.8.3 workflow and visualized using the web-based tool iTOL v6. (**B**) Heatmap of whole genome-based average nucleotide identity (ANI) of the 24 *B. cereus* group isolates and other type strains of *B. cereus* group genome sequences, constructed using FastANI v1.33.

The sources and genomic information of the 24 *B. cereus* group isolates investigated in this study are summarized in Table 1. A phylogenetic tree of the 24 *B. cereus* group isolates and other type strains of *B. cereus* group genome sequences was generated using GtoTree, a genome-based phylogenomics workflow (Figure 1A,B).

Based on this phylogenetic tree, the 24 *B. cereus* group isolates were classified as *B. cereus s.s.* (12 isolates), *B. pacificus* (5 isolates), *B. paranthracis* (5 isolates), *B. tropicus* (1 isolate), and “*B. bingmayongensis*” (1 isolate). In detail, *Bacillus* sp. B08, *Bacillus* sp. B14, *Bacillus* sp. B21, *Bacillus* sp. B54, *Bacillus* sp. B66, *Bacillus* sp. B68, *Bacillus* sp. B69, *Bacillus* sp. B102, *Bacillus* sp. B112, *Bacillus* sp. B123, *Bacillus* sp. B124, and *Bacillus* sp. B125 were closely related to *B. cereus s.s.*; *Bacillus* sp. B18, *Bacillus* sp. B62, *Bacillus* sp. B92, *Bacillus* sp. B106, and *Bacillus* sp. B122 were closely related to *B. pacificus*; *Bacillus* sp. B81, *Bacillus* sp. B84, *Bacillus* sp. B85, *Bacillus* sp. B94, and *Bacillus* sp. B98 were closely related to *B. paranthracis*; *Bacillus* sp. B134 was closely related to *B. tropicus*; and *Bacillus* sp. B103 was closely related to “*B. bingmayongensis*”.

**Figure 2:**
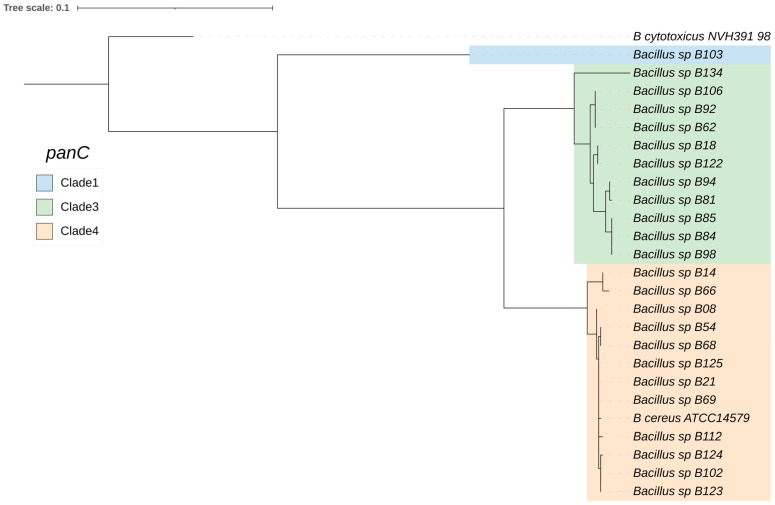
Maximum likelihood core-gene phylogenetic tree of the 24 *B. cereus* group isolates. The tree was constructed using *panC* sequences extracted from the BTyper3 tool in RAxML, with the GTRGAMMA model and 1000 bootstrap replicates. The clades were designated according to previously proposed phylogenetic groups based on *panC* sequence types.

Next, the genomic sequences of the 24 *B. cereus* group isolates were used to generate a core-gene phylogenetic tree based on *panC* (Figure 2). This revealed the following three *B. cereus* group clades (designated according to previously proposed phylogenetic groups based on *panC* sequence types [28]): clade III (11 isolates), clade IV (12 isolates, which is the same clade as *B. cereus s.s.*), and clade I (1 isolate, which was *Bacillus* sp. B103, closely related to “*B. bingmayongensis*”).

### 2.2. Subsystem Categorization

**Figure 3:**
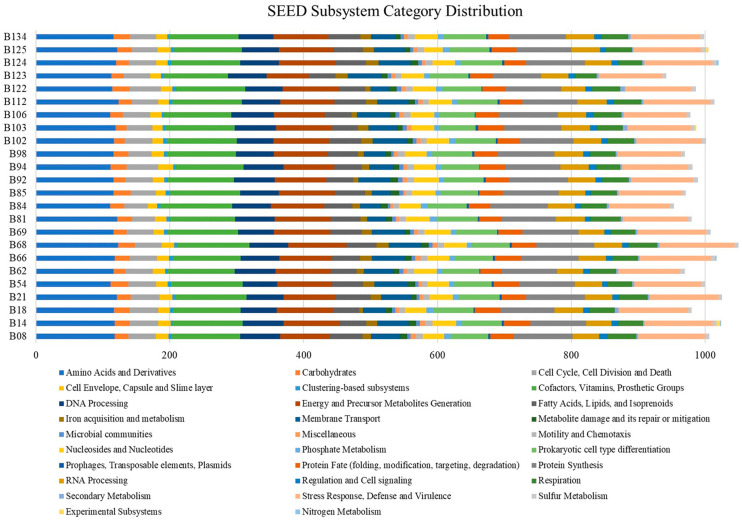
Comparison of functional categories in the *B. cereus* isolate genomes based on SEED subsystem categorization. Functional categorization was based on the roles of annotated and assigned genes. Each colored bar denotes the number of genes assigned to each category.

The SEED subsystem categorization based on RASTtk annotation is illustrated in Figure 3. The analysis predicted 27–29 categories among the 24 *B. cereus* group genomes. The most abundant systems were “Amino Acids and Derivatives”, followed by “Cofactors, Vitamins, Prosthetic Groups” and “Stress Response, Defense, and Virulence”. In contrast, the genomes had a remarkably low number of “Prophages, Transposable Elements, Plasmids” and “Secondary Metabolism”. Several of the isolates exhibited “Experimental Subsystems” and “Nitrogen Metabolism” in their genomes.

### 2.3. AMR Gene Analysis

**Figure 4:**
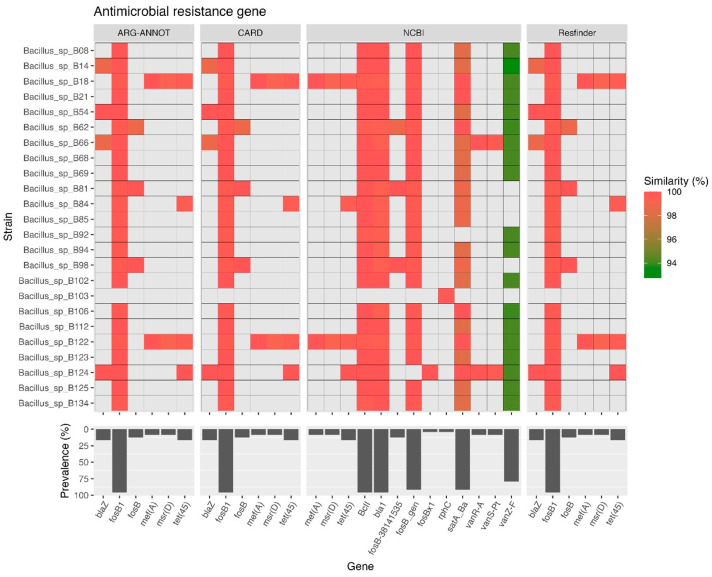
Distribution of AMR genes among the *B. cereus* group isolates. The presence of AMR genes in the draft genomes of the isolates was determined using ABRicate v0.8, which involved the ARG-ANNOT, CARD, NCBI, and ResFinder databases. The prevalence of each gene is shown as a percentage. *blaZ*: beta-lactamase III gene, *fosB1*: fosfomycin resistance gene, *fosB*: fosfomycin resistance gene, *mef(A)*: macrolide efflux gene, *msr(D)*: macrolide resistance gene, *tet(45)*: tetracycline resistance gene, *BcII*: beta-lactamase gene, *bla1*: beta-lactam resistance gene, *rphC*: rifampicin resistance gene, *satA_Ba*: streptothricin resistance gene, *vanR-A*: glycopeptide resistance gene, *vanS-Pt*: glycopeptide resistance gene, *vanZ-F*: glycopeptide resistance gene.

AMR genes in the *B. cereus* group isolate genomes were identified (Figure 4). AMR genes that are associated with resistance to one or more antimicrobials were predicted based on the ARG-ANNOT, CARD, NCBI, and ResFinder databases. The data from ARG-ANNOT, CARD, and ResFinder databases all showed that *fosB1* (95.8%) was the most prevalent gene among the six observed AMR genes. The other five AMR genes were *blaZ* (16.7%), *fosB* (12.5%), *mef(A)* (8.3%), *msr(D)* (8.3%), and *tet(45)* (16.7%). The NCBI database showed that there were 13 AMR genes, with *bcII* (95.8%) and *bla1* (95.8%) being the most prevalent, followed by *fosB_gen* (91.7%), *satA_Ba* (encoding streptothricin-N-acetyltransferase) (91.7%), *vanZ-F* (79.2%), *tet(45)* (16.7%), *fosB-38141535* (12.5%), *mef(A)* (8.3%), *msr(D)* (8.3%), *vanR-A* (8.3%), *vanS-Pt* (8.3%), *rphC* (encoding rifamycin-inactivating phosphotransferase) (4.2%), and *fosBx1* (4.2%).

### 2.4. Virulence Factor Gene Analysis

**Figure 5:**
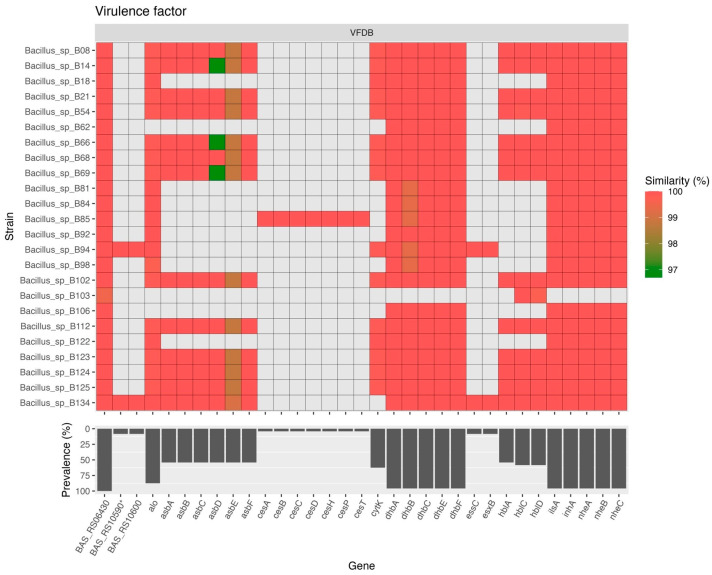
Distribution of virulence genes among the *B. cereus* group isolates. The putative virulence factors were predicted by a BLAST search against the Virulence Factor Database. The prevalence of each gene is shown as a percentage. *alo*: thiol-activated cytolysin, *asb*: *B. anthracis* siderophore biosynthesis, *ces*: cereulide synthetase, *cytK*: cytolytic pore-forming protein cytotoxin K, *dhb*: bacillibactin biosynthesis, *essC*: type VII secretion protein, *esxB*: WXG100 proteins, *hblA*: hemolysin BL binding component precursor, *hblC*: hemolysin BL lytic component L2, *hblD*: hypothetical protein, *ilsA*: iron-regulated leucine rich surface protein, *inhA*: immune inhibitor A, metalloprotease, *nheA*: non-hemolytic enterotoxin A, *nheB*: non-hemolytic enterotoxin lytic component L1, *nheC*: enterotoxin C.

There were 33 genes in the *B. cereus* group isolate genomes that were classified as virulence factors according to the VFDB database (Figure 5). Among these genes, the most prevalent gene (harbored by all 24 isolates) was *BAS_RS06430* (*inhA1* in *Bacillus anthracis* str. *Sterne*; complete sequence available at https://www.genome.jp/dbget-bin/www_bget?refseq+NC_005945, unpublished data, accessed on 12 January 2024). Additionally, 23 (95.8%) of the isolates harbored bacillibactin biosynthesis genes (*dhbA*, *dhbB*, *dhbC*, *dhbE*, and *dhbF*), genes encoding the three-component non-hemolytic enterotoxin (*nheA*, *nheB*, and *nheC*), a gene encoding an iron-regulated leucine-rich surface protein (*ilsA*), and a gene encoding a metalloprotease (*inhA*). Moreover, 21 (87.5%) and 15 (62.5%) of the isolates harbored genes encoding cytotoxin anthrolysin O (*alo*), which is also known as a cholesterol-dependent cytolysin secreted by *B. anthracis*, and the single-component enterotoxin cytotoxin K (*cytK*), respectively. Furthermore, 13–14 (54.2–58.3%) of the isolates harbored genes encoding the three-component enterotoxin hemolysin BL (*hblA*, *hblC* and *hblD*), which was similar to the prevalence of genes (13 [54.2%]) in the *B. anthracis* siderophore biosynthesis (*asb*) operon (*asbA*, *asbB*, *asbC*, *asbD*, *asbE,* and *asbF*). Only two (8.3%) isolates harbored BAS_RS10590*, *BAS_RS10600*, type VII secretion gene (*essC*), and WXG100 gene (*esxB*). Only one (4.2%) isolate harbored a cereulide synthetase (*ces*) gene cluster (*cesA*, *cesB*, *cesC*, *cesD*, *cesH*, *cesP*, and *cesT*).

### 2.5. Biofilm-Associated Gene Analysis

**Figure 6:**
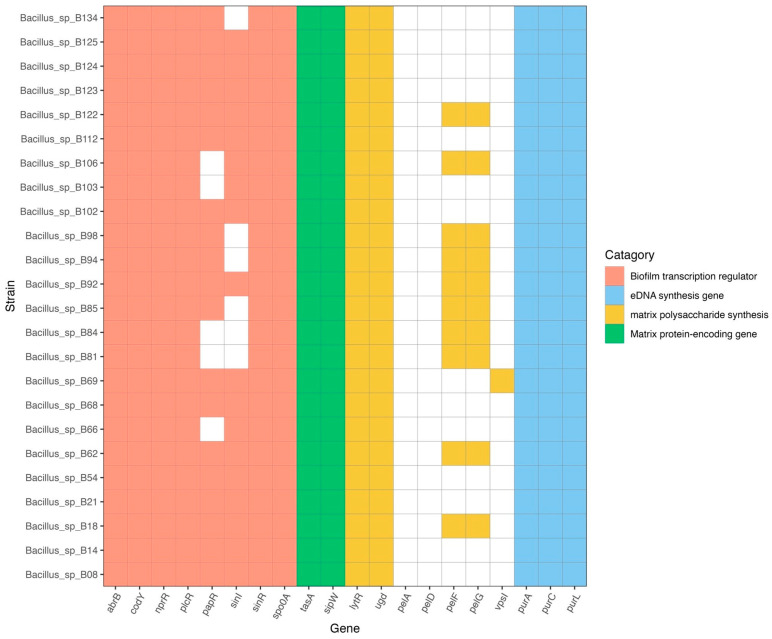
Distribution of biofilm-associated genes among the 24 B. cereus group isolates. Genes relevant to biofilm formation (biofilm transcription regulator genes, eDNA synthesis genes, matrix polysaccharide synthesis genes, and matrix protein-encoding genes) were identified using BlastKOALA.

The biofilm-associated genes in the *B. cereus* group genomes were identified using BlastKOALA (https://www.kegg.jp/blastkoala/, accessed on 3 November 2023) (Figure 6). The crucial genes for biofilm formation comprise matrix protein-encoding genes, matrix polysaccharide synthesis genes, eDNA synthesis genes, and biofilm transcription regulator genes. All isolates harbored *tasA* and *sipW* genes (matrix protein-encoding genes); *purA*, *purC*, and *purL* genes (eDNA synthesis genes); *lytR* and *ugd* genes (matrix polysaccharide synthesis genes); and *abrB*, *codY*, *nprR*, *plcR*, *sinR*, and *spo0A* genes (biofilm transcription regulator genes). Additionally, 19 (79%) isolates harbored *papR* (biofilm transcription regulator gene), while 18 (75%) isolates harbored *sinI* (another biofilm transcription regulator gene). Moreover, 10 (41.7%) isolates harbored *pelF* and *pelG* of the *pel* operon (which encodes matrix polysaccharide synthesis genes), but none harbored *pelA* or *pelD*. Only one (4.2%) isolate (*Bacillus* sp. B69) harbored *vpsI* (exopolysaccharide synthesis gene, to produce the main component of the biofilm matrix).

## 3. Discussion

The *Bacillus cereus* group, also known as *B. cereus s.l.*, is composed of several species, some of which can cause gastrointestinal or non-gastrointestinal tract infections. Members of the *B. cereus* group are classified into seven phylogenetic clades based on *panC*: clade I, *B. pseudomycoides*; clade II, *B. wiedmannii*; clade III, *B. anthracis*; clade IV, *B. cereus s.s.* and *B. thuringiensis*; clade V, *B. toyonensis*; clade VI, *B. weihenstephanensis* and *B. mycoides*; and clade VII, *B. cytotoxicus* [28,29,30]. In this study, 24 *B. cereus* group isolates from foodstuffs were characterized using WGS and bioinformatic tools. Most isolates were phylogenetically classified as *B. cereus s.s.* (clade IV, 12 isolates) followed by *B. pacificus* (clade III, 5 isolates), *B. paranthracis* (clade III, 5 isolate), *B. tropicus* (clade III, 1 isolate), and “*B. bingmayongensis*” (clade I, 1 isolate).

*B. cereus s.s.* is a serious human pathogen that has caused foodborne outbreaks in several countries including Thailand. Most cases involved self-limiting diarrhea, nausea, or vomiting due to bacterial enterotoxins, especially hemolysin BL (HBL), the three-component non-hemolytic enterotoxin (NHE), and cytotoxin K (CytK), as well as cereulide [31]. In this study, 12 isolates were closely related to *B. cereus s.s*. Furthermore, 23 (95.8%) of the 24 *B. cereus* group isolates (all isolates except for *Bacillus* sp. B103, which was closely related to “*B. bingmayongensis*”) harbored *nhe* genes. However, the presence of toxin genes is not always consistent with the pathogenic potential of a *B. cereus* group isolate [29]. Moreover, BTyper may detect the presence of a toxin gene, while the PCR result may be negative [29]. A negative PCR result may be due to variant sequences causing poor binding of the published primers to the DNA sequence [32]. Therefore, cytotoxicity assays of *B. cereus* group isolates, along with their virulence factor gene profiles, may offer more information about potential harm to human health.

*B. pacificus* was isolated from the sediment of the Pacific Ocean [9], and, recently, the *B. pacificus* strain CR121 was isolated from the intestine of the Indian major carp species rohu (*Labeo rohita*) [33]. The CR121 strain has been proposed to be a candidate fish probiotic as it possesses *in vivo* disease prevention efficacy in *L. rohita* and *Oreochromis niloticus* [33]. Moreover, a *B. pacificus* strain was isolated from a spicy mussel salad in Pathum Thani province, Thailand, which was concerning as it exhibited strong biofilm formation and tetracycline resistance phenotypes [27]. In this study, five isolates were closely related to *B. pacificus*, comprising *Bacillus* sp. B18 (isolated from stir-fried mixed vegetables), *Bacillus* sp. B62 (isolated from rice with spicy shrimp paste dip and fried mackerel), *Bacillus* sp. B92 (isolated from sushi), *Bacillus* sp. B106 (isolated from stir-fried lotus stem and straw mushroom with pork), and *Bacillus* sp. B122 (isolated from stir-fried pickled turnip with egg). It is suggested that *B. pacificus* can be isolated from foodstuffs that do not contain seafood. In addition, contamination may occur during or after-cooking processing, which should be considered in more depth.

*B. paranthracis*, which was first described in 2017 [9], has been identified as group III *B. cereus* according to microbiologic methods and *panC* phylogenetic group assignment [34]. In 2020, *B. paranthracis* was isolated from blood of a fatal Ebola virus disease case in Liberia and was identified by whole genome sequencing [35]. *B. paranthracis* is considered as a pathogenic bacterium both in human and animal hosts since it could be isolated from the milk of cows diagnosed with mastitis [36]. It is also known as emetic *B. cereus* due to the ability to produce cereulide, an emetic toxin. In 2016, *B. paranthracis* was linked to a foodborne outbreak, possibly caused by the consumption of refried beans, in upstate New York [37]. Five isolates in this study were closely related to *B. paranthracis*, comprising *Bacillus* sp. B81 (isolated from spicy Vietnamese pork sausage salad), *Bacillus* sp. B84 (isolated from spicy tremella mushroom salad), *Bacillus* sp. B85 (isolated from spicy pork meatball salad), *Bacillus* sp. B94 (isolated from spicy fermented fish dip), and *Bacillus* sp. B98 (isolated from stir-fried glass noodles). Most of the contaminated food samples were spicy salad. The ingredients of this type of food might be further determined.

*B. bingmayongensis* strain FJAT-13831 was originally isolated from the pit soil of Emperor Qin’s terracotta warriors in China [38]. The colonies on nutrient agar were flat, greyish white, undulate in margin. Its closest phylogenetic neighbor was *B. pseudomycoides*. In 2022, *B. bingmayongensis* KNUAS006 was isolated from fermented Korean food samples and classified as a probiotic strain [39]. Currently, little is known about the pathogenic potential of both *B. bingmayongensis* and *B. pseudomycoides*. Literally, *B. pseudomycoides*, characterized by rhizoidal colonies on culture media [3], is not recognized as a pathogen, but its toxigenic potential remains uncertain. A previous study reported that all *B. pseudomycoides* isolates harbor *hblA* [40], another study reported that 30% and 70% of *B. pseudomycoides* isolates harbor *nheA* and *hblA*, respectively [41], and a third study reported that a *B. pseudomycoides* isolate (isolated from Shepherd’s purse in South Korea) harbored all the genes of the HBL enterotoxin complex (*hblA*, *hblC*, and *hblD*) [42]. The presence of enterotoxin genes seems to vary among isolates in various studies. In this study, we found that *Bacillus* sp. B103 (isolated from salad roll with crab stick and exhibiting rhizoidal colonies) was closely related to *B. bingmayongensis*. The isolate harbored three virulence genes comprising *BAS_RS06430*, *hblC,* and *hblD* genes. Therefore, a safety assessment of *B. bingmayongensis* needs to be performed. Moreover, the colony morphology of *Bacillus* sp. B103 exhibited rhizoidal appearance while it was classified as *B. bingmayongensis*. However, the ANI value of *Bacillus* sp. B103 was only 90.05%, which is below the 95% cutoff value for the delimitation of bacterial species. Therefore, species classification within the *B. cereus* group is still challenging.

*B. tropicus* has been isolated from various environmental samples, including a soil sample from a marine duck farm of Beibu Gulf in Guangxi, China [43]; a soil sample from a city center rubbish dump in Durgapur, India [44]; and kawal (a fermented condiment obtained by natural and alkaline fermentation of *Senna obtusifolia* leaves) from the markets of Abéché, Bokoro, Mandelia, and N’Djamena in Tchad [45]. In this study, one isolate (*Bacillus* sp. B134, isolated from stewed pork leg on rice) was closely related to *B. tropicus*. The isolate harbored several virulence genes including *nhe* and *hbl* but not *cytK*. In addition, *essC* and *esxB* were present. Esat-6 protein secretion systems (ESX or Ess) are required for the virulence of several human pathogens including *Mycobacterium tuberculosis* and *Staphylococcus aureus* as well as *B. subtilis* [46]. However, the molecular mechanism of this system in relation to the virulence of *B. tropicus* needs to be further characterized.

Determining the prevalence of antimicrobial resistance among *B. cereus* group isolates can provide information for proper antibiotic use and improve treatment outcomes [24]. Although most *B. cereus* group infections tend to be self-limiting and may not need antimicrobial treatment, severe infections, particularly in immunocompromised patients, need to be treated with appropriate antibiotic agents. The most suitable drug of choice for *B. cereus* bacteremia is vancomycin [47]. Moreover, carbapenem antibiotics are also considered to be an effective treatment [15]. *B. cereus s.s.* is typically resistant to beta-lactam antibiotics [48]. In addition, acquired resistance to commonly used antibiotics such as ciprofloxacin, cloxacillin, erythromycin, tetracycline, and streptomycin has been literally reported [48,49]. The AMR mechanisms of *B. cereus* group strains may vary between strains [50]. In this study, all isolates except for *Bacillus* sp. B103 harbored *bcII* and *bla1* (related to resistance to beta-lactams) and *fosB1* (related to the inactivation of fosfomycin antibiotic). Minimum inhibitory concentration (MIC) experiments of *B. cereus* isolates (from indoor air) that harbored *fosB*, *bcI*, and *bcII* have demonstrated their ampicillin and fosfomycin resistance phenotypes [50]. Other AMR genes were observed in some of our isolates, including those related to resistance to tetracycline and vancomycin. The results of AMR gene analysis in this study were consistent with antimicrobial susceptibility testing in the previous study [51]. The isolates that harbored genes responsible for beta-lactam resistance showed the resistance phenotypes to ampicillin, amoxicillin-clavulanic acid, and penicillin. Two isolates (*Bacillus* sp. B18 and *Bacillus* sp. B122) which harbored genes responsible for macrolide resistance (*mef(A)* and *msr(D)*) demonstrated intermediate resistance to erythromycin. However, discrepancies between antimicrobial resistance phenotypes and the prevalence of antimicrobial resistance genes were observed in this study. The isolates that carried the *tet(45)* gene exhibited a susceptible phenotype to tetracycline. It has been reported that *Bacillus* isolates showed an antibiotic phenotype that was sensitive to tetracycline while the *tetB* detection rate was 100% [52]. This might be due to the presence of varied antimicrobial resistance mechanisms and many genetic factors required for the resistance phenotypes [53]. Moreover, the AMR phenotypes to the other antimicrobial agents (e.g., fosfomycin) need to be further determined in vitro. In addition, the transferable mobile genetic elements (such as transposon or insertion sequences) should be characterized based on long-read genome sequencing in order to verify the risk of horizontal transfer of AMR genes among bacterial species [54].

Among the virulence genes observed in this study, the most prevalent gene (harbored by all 24 isolates) was *BAS_RS06430* (*inhA1* in *Bacillus anthracis* str. *Sterne*). It has been reported that *inhA1* is produced only by pathogenic members of the *Bacillus* genus and associated with the cleavage of host proteins during infection [55]. Moreover, 95.8% of the isolates harbored a gene encoding a metalloprotease (*inhA*). Therefore, the pathogenic effects of these genes should be further characterized. Additionally, most isolates (95.8%) harbored bacillibactin biosynthesis genes (*dhbA*, *dhbB*, *dhbC*, *dhbE*, and *dhbF*), genes encoding the three-component non-hemolytic enterotoxin (*nheA*, *nheB*, and *nheC*), and a gene encoding an iron-regulated leucine-rich surface protein (*ilsA*). Under iron-limited conditions, *B. anthracis* produces two catecholate siderophores which are petrobactin (encoded by *asb* operon) and bacillibactin (encoded by *bac* operon) [56,57]. However, only the *asb* locus is essential for growth in iron-depleted media and for virulence in mice [56]. Daou et al. [58] reported that IlsA-like proteins are restricted to *B. cereus* group bacteria. IlsA is an important adaptation factor required for the development of *B. cereus* in susceptible hosts including mammals and insects [58]. The isolates that harbored these virulence genes are of interest since they may cause harmful effects to the human host in some circumstances.

Biofilm formation is a potential virulence determinant of various pathogens including *B. cereus* group species as it allows the bacteria to resist disinfectants and antimicrobials, as well as to escape the host immune system [59,60]. The important genes for biofilm formation in *B. cereus* group species include biofilm transcriptional regulator genes (*abrB*, *codY*, *nprR*, *plcR*, *papR*, *sinI*, *sinR*, and *spo0A*), matrix protein-encoding genes (*tasA* and *sipW*), matrix polysaccharide synthesis genes (*lytR*, *ugd*, *vpsI,* and *pelDEA_DA_FG* operon), eDNA synthesis genes (*purA*, *purC*, and *purL*), and cyclic-di-GMP metabolism genes [27,61]. All isolates in this study harbored the above-mentioned biofilm-associated genes except for *papR*, *sinI,* and the *pelDEA_DA_FG* operon, which were present in some isolates. Moreover, *vpsI* (exopolysaccharide synthesis gene) was found in one isolate (*Bacillus* sp. 69). In *Vibrio cholerae*, the biofilm matrix is mainly composed of exopolysaccharides (VPS), whose synthesis is encoded by two *vps* operons (*vpsI* and *vpsII*) [62]. The role of *vpsI* in biofilm formation in *Bacillus* species is still unclear. It has been proposed that the *pelDEA_DA_FG* operon might play an important role in the biofilm-formation capacity of *Bacillus* species, as the lack of this operon reduced the biofilm-formation capacity of the tested strain [27,63]. Comprehensive *in vitro* biofilm formation assays need to be performed to confirm the potential roles of these genes in biofilm formation in *Bacillus* species.

In conclusion, we employed WGS to characterize 24 *B. cereus* group isolates from foodstuffs. We identified the closest reference strains to the isolates based on a phylogenetic tree analysis and whole genome-based average nucleotide identity. The most prevalent species were *B. cereus s.s.* (12 isolates) followed by *B. pacificus* (5 isolates). The remaining three species were *B. paranthracis*, *B. tropicus*, and *B. bingmayongensis*. We also observed the prevalence of AMR and virulence genes in the genomes of the isolates. Most isolates harbored genes related to resistance to fosfomycin and beta-lactams. Moreover, additional AMR genes were identified in some isolates, including those related to resistance to erythromycin and tetracycline. However, the risk of horizontal transfer of AMR genes among *B. cereus* group species and other bacterial species needs to be further clarified. Most of the isolates harbored virulence genes (especially those related to metalloprotease production, bacillibactin biosynthesis, and enterotoxin production). Altogether, our findings suggest that WGS combined with appropriate bioinformatic tools can be used to evaluate the pathogenic potential of *B. cereus* group isolates from foodstuffs. Determining the genetic epidemiology, the presence of virulence and AMR genes, and the potential hazards of foodborne *B. cereus* group species help to raise awareness of the underestimated foodborne diseases caused by these species.

## 4. Materials and Methods

### 4.1. Bacterial Strains

Twenty-four *B. cereus* group isolates from foodstuffs collected in 2018 [51] in Pathum Thani Province, Thailand, were characterized in this study. The sampling procedure and microbiological analysis of the isolates has been previously described [51]. Briefly, all isolates were identified using both conventional microbiological techniques and API 50 CHB test. Glycerol stocks of the bacteria (stored at −80 °C) were cultured on nutrient agar plates and incubated overnight at 35 ± 2 °C.

### 4.2. DNA Extraction and WGS

The genomic DNA was extracted using Monarch HMW DNA Extraction Kits (NEB, Ipswich, MA, USA) according to the manufacturer’s instructions. The purified genomic DNA was qualitatively assessed using a fluorometer. Paired-end sequencing (2 × 250-bp reads) was performed using V2 chemistry and a MiSeq platform (Illumina, San Diego, CA, USA). The reads were processed using Trimmomatic v0.39 to remove adapter and barcode sequences, correct mismatched bases in overlaps, and filter out low-quality reads (<Q30) [64]. FastQC v0.12.1 was used to determine the quality of the reads.

### 4.3. Genome Assembly and Annotation

Assembly of the short-read data sets was performed using Unicycler v0.5.0 with default parameters [65]. QUAST v5.2.0 was used to evaluate the genome assembly quality [66]. FastANI v1.33 was used to calculate the genome-wide average nucleotide identity (ANI) between genomes [67]. Gene prediction and annotation were performed using Prokka v1.14.6 [68] and Bacterial and Viral Bioinformatics Resource Center (BV-BRC) (https://www.bv-brc.org/, accessed on 12 September 2023) [69]. Functional annotation of the predicted proteins was conducted using BlastKOALA of Kyoto Encyclopedia of Genes and Genomes (KEGG) (https://www.kegg.jp/blastkoala/, accessed on 3 November 2023) [70].

The presence of AMR genes in the draft genomes of the strains was determined using ABRicate v0.8 (https://github.com/tseemann/ABRicate, accessed on 15 September 2023), which included several predownloaded databases (ResFinder [71], CARD [72], NCBI [73], ARG-ANNOT [74], and NDARO [75]) with default settings [76]. Putative virulence factors were predicted by conducting BLAST searches against the Virulence Factor Database (VFDB) (http://www.mgc.ac.cn/VFs/main.htm, accessed on 15 September 2023) [77]. BTyper3 v3.4.0 was used to assign each genome, determine the multilocus sequence typing (MLST) profiles, and identify the *panC*-based phylogenetic groups [78].

### 4.4. Phylogenetic Trees

A maximum likelihood phylogenetic tree of the 24 *B. cereus* group isolates and other type strains of *B. cereus* group genome sequences obtained from NCBI was constructed. This involved using GToTree v1.8.3 with the hidden Markov model (HMM) source bacteria and default parameters [79].

Next, the *panC* nucleotide sequences of the 24 *B. cereus* group isolates were extracted from the BTyper3 tool [78] and then aligned using the multiple sequence alignment algorithm MUSCLE [80]. A maximum likelihood core-gene phylogenetic tree was constructed using RAxML with the GTRGAMMA model and 1000 bootstrap replicates. The phylogenetic tree was visualized using iTOL v6 (https://itol.embl.de/, accessed on 17 October 2023).

### 4.5. Subsystem Categorization

The 24 *B. cereus* group genomes were submitted to Rapid Annotations using Subsystems Technology (RASTtk), available in The Pathosystems Resource Integration Center (PATRIC) (https://www.bv-brc.org/ accessed on 12 September 2023), for annotation, subsystem categorization, and comparison purposes [81].

### 4.6. Nucleotide Sequence Accession Numbers

The draft genomes of the 24 *B. cereus* group isolates were deposited in the NCBI database (https://submit.ncbi.nlm.nih.gov/subs/, accessed on 18 October 2023) under the accession numbers shown in Table 1. The GenBank BioProject ID is PRJNA1030778.

## Figures and Tables

**Table 1:** The genomes of *Bacillus* spp. used in this study.

Strain Names	Source	BioSample Accession	Closest species (ANI%)	Coverage	Contig (s)	Size (bp)	GC%	CDS
B08	spicy green papaya salad with bamboo shoot	SAMN37915793	*B. cereus* (98.84%)	78.2×	39	5,344,844	35.06	5371
B14	clear soup with tofu and minced pork	SAMN37915794	*B. cereus* (96.86%)	57.8×	258	6,378,095	34.66	6524
B18	stir-fried mixed vegetables	SAMN37915795	*B. pacificus* (99.92%)	74.1×	55	5,453,845	35.27	5571
B21	stir-fried pumpkin	SAMN37915796	*B. cereus* (99.85%)	81.8×	30	5,262,391	35.04	5292
B54	stir-fried noodle with yentafo sauce	SAMN37915797	*B. cereus* (98.67%)	68.4×	34	5,880,551	34.80	5970
B62	rice with spicy shrimp paste dip and fried mackerel	SAMN37915798	*B. pacificus* (98.12%)	72.5×	145	5,542,779	35.17	5703
B66	stir-fried noodle	SAMN37915799	*B. cereus* (96.98%)	61.4×	104	6,152,935	34.76	6074
B68	stir-fried mixed vegetables	SAMN37915800	*B. cereus* (98.75%)	73.1×	65	5,951,707	35.01	6082
B69	spicy mixed vegetable soup	SAMN37915801	*B. cereus* (98.90%)	71.7×	43	5,378,133	35.01	5376
B81	spicy Vietnamese pork sausage salad	SAMN37915802	*B. paranthracis* (97.80%)	73.4×	44	5,223,900	35.35	5287
B84	spicy tremella mushroom salad	SAMN37915803	*B. paranthracis* (97.68%)	80.9×	52	5,126,336	35.34	5169
B85	spicy pork meatball salad	SAMN37915804	*B. paranthracis* (97.54%)	77.9×	78	5,473,500	35.25	5546
B92	sushi	SAMN37915805	*B. pacificus* (98.14%)	70.9×	100	5,528,812	35.31	5660
B94	spicy fermented fish dip	SAMN37915806	*B. paranthracis* (98.52%)	66.2×	60	5,435,642	35.15	5496
B98	stir-fried glass noodles	SAMN37915807	*B. paranthracis* (97.53%)	157.5×	97	5,426,062	35.17	5512
B102	sandwich	SAMN37915808	*B. cereus* (98.47%)	188.0×	37	5,221,584	35.08	5237
B103	salad roll with crab stick	SAMN37915809	“*B. bingmayongensis*” (90.05%)	181.6×	64	5,437,995	35.50	5436
B106	stir-fried lotus stem and straw mushroom with pork	SAMN37915810	*B. pacificus* (98.18%)	76.8×	70	5,401,240	35.18	5541
B112	stir-fried tofu with bean sprouts	SAMN37915811	*B. cereus* (98.87%)	122.2×	127	5,712,866	35.05	5773
B122	stir-fried pickled turnip with egg	SAMN37915812	*B. pacificus* (99.95%)	64.5×	61	5,431,860	35.27	5487
B123	stir-fried zucchini with egg	SAMN37915813	*B. cereus* (98.85%)	66.2×	47	5,481,273	34.87	5454
B124	egg noodles with roasted red pork and wonton	SAMN37915814	*B. cereus* (98.89%)	89.7×	58	5,856,371	34.76	5803
B125	Thai rice noodle salad roll	SAMN37915815	*B. cereus* (98.85%)	84.3×	27	5,631,595	34.92	5647
B134	stewed pork leg on rice	SAMN37915816	*B. tropicus* (96.87%)	47.7×	38	5,208,045	35.24	5239

ANI: Average Nucleotide Identity, GC: Guanine and Cytosine, CDS: Coding Sequence.

## Data Availability

Data are contained within the article.
